# RETRACTED: Yang et al. Capacitive Behavior of Aqueous Electrical Double Layer Based on Dipole Dimer Water Model. *Nanomaterials* 2023, *13*, 16

**DOI:** 10.3390/nano15010001

**Published:** 2024-12-24

**Authors:** Songming Yang, Youer Deng, Shiqi Zhou

**Affiliations:** 1 School of Physics and Electronics, Central South University, Changsha 410083, China; yang-sm20@mails.tsinghua.edu.cn (S.Y.); dumbledoresky@gmail.com (Y.D.); 2 Zhili College, Tsinghua University, Beijing 100084, China

The journal retracts the article titled “Capacitive Behavior of Aqueous Electrical Double Layer Based on Dipole Dimer Water Model” [1], cited above.

Following publication, the authors contacted the Editorial Office to raise concerns relating to using the dipole-dimer model to represent water molecules in electrolyte aqueous solutions for supercapacitors. 

Adhering to our standard procedure, the Editorial Office and Editorial Board conducted an investigation that confirmed that results for the dipole-dimer water model are not reliable. The relative dielectric constant was arbitrarily set to 6.0 in the calculations. Considering that the value of the relative dielectric constant in the double-layer capacitor would affect the size and behavior of the differential capacitance, the arbitrary assumption of the relative dielectric constant being 6.0 in the calculation makes the calculation results arbitrary and not universally applicable. The model’s assumptions, beyond an arbitrary “residual” permittivity of 6.0 that lacks experimental or simulation-based validation, also include arbitrary parameters such as a 1.9 Å diameter and a polarization charge of 0.203947*e* at two sites, which are similarly unsupported by empirical or computational evidence. These assumptions result in significant discrepancies between theoretical predictions and the actual system pressures under the conditions analyzed in this article. For instance, as shown in Figure 1 [1], for electrolyte concentrations of 0.25 M, 1.0 M, 4.0 M, and 6.0 M, the model predicts bulk pressures (in standard atmospheric units) of 5546, 5742, 6537, and 7164, respectively. At 298.15 K, Figure 3 [1] shows a predicted bulk pressure of 5546. Likewise, for cation diameters (in Å) of 2, 2.4, 2.8, 3.2, 3.6, and 4, Figure 4 [1] predicts bulk pressures of 5564, 5801, 6117, 6537, 7094, and 7842, respectively. All these values are far greater than one atmosphere, demonstrating that the calculations are arbitrary, lack universal applicability, and are inconsistent with the behavior of real aqueous electrolytes. It was only during further investigations conducted after the publication of the article that the authors discovered the significant discrepancy between the theoretically predicted pressures and the real atmospheric pressure, as the publication of this article [1] did not necessitate verifying the magnitude of the theoretical pressures. This realization led them to understand that the dipole-dimer model employed in their calculations is fundamentally unreliable as a representation of water.

As a result, the Editorial Office, Editorial Board, and authors have decided to retract this article [1] as per MDPI’s retraction policy (https://www.mdpi.com/ethics#_bookmark30, accessed on 5 November 2024).

This retraction was approved by the Editor-in-Chief of the *Nanomaterials* journal.

The authors agreed to this retraction.
